# Effect of Cremophor RH40, Hydroxypropyl Methylcellulose, and Mixing Speed on Physicochemical Properties of Films Containing Nanostructured Lipid Carriers Loaded with Furosemide Using the Box–Behnken Design

**DOI:** 10.3390/polym16111605

**Published:** 2024-06-05

**Authors:** Pakorn Kraisit, Namon Hirun, Premjit Limpamanoch, Yongthida Sawaengsuk, Narumol Janchoochai, Ornpreeya Manasaksirikul, Sontaya Limmatvapirat

**Affiliations:** 1 Thammasat University Research Unit in Smart Materials and Innovative Technology for Pharmaceutical Applications (SMIT-Pharm), Faculty of Pharmacy, Thammasat University, Pathumthani 12120, Thailand; namon.hi@tu.ac.th (N.H.); premjit1993@hotmail.com (P.L.); 2 Division of Pharmaceutical Sciences, Faculty of Pharmacy, Thammasat University, Pathumthani 12120, Thailand; yongthida.s@gmail.com (Y.S.); niienan@hotmail.com (N.J.); rneetx@gmail.com (O.M.); 3 Pharmaceutical Biopolymer Group (PBiG), Faculty of Pharmacy, Silpakorn University, Nakhon Pathom 73000, Thailand; limmatvapirat_s@su.ac.th

**Keywords:** Cremophor RH40, hydroxypropyl methylcellulose (HPMC), nanostructured lipid carriers (NLCs), films, Box–Behnken design, furosemide

## Abstract

This study aimed to examine the characteristics of H-K4M hydroxypropyl methylcellulose (HPMC) films containing nanostructured lipid carriers (NLCs) loaded with furosemide. A hot homogenization technique and an ultrasonic probe were used to prepare and reduce the size of the NLCs. Films were made using the casting technique. This study used a Box–Behnken design to evaluate the influence of three key independent variables, specifically H-K4M concentration (X_1_), surfactant Cremophor RH40 concentration (X_2_), and mixing speed (X_3_), on the physicochemical properties of furosemide-loaded NLCs and films. The furosemide-loaded NLCs had a particle size ranging from 54.67 to 99.13 nm, and a polydispersity index (PDI) ranging from 0.246 to 0.670. All formulations exhibited a negative zeta potential, ranging from −7.05 to −5.61 mV. The prepared films had thicknesses and weights ranging from 0.1240 to 0.2034 mm and 0.0283 to 0.0450 g, respectively. The drug content was over 85%. Film surface wettability was assessed based on the contact angle, ranging from 32.27 to 68.94°. Film tensile strength varied from 1.38 to 7.77 MPa, and their elongation at break varied from 124.19 to 170.72%. The ATR-FTIR analysis confirmed the complete incorporation of the drug in the film matrix. Therefore, the appropriate selection of values for key parameters in the synthesis of HPMC films containing drug-loaded NLCs is important in the effective development of films for medical applications.

## 1. Introduction

The delivery of drugs or active agents to specific organs or targets of interest has been a major challenge for researchers, leading to the development of numerous novel drug delivery system formats, particularly those in which the particles are in the nanometer size range. Indeed, lipid nanoparticles (LNPs) have emerged as a promising platform. Early iterations of this approach involved solid lipid nanoparticles (SLNs) composed primarily of solid lipids, stabilizers, or surfactants with the active agent encapsulated within the particle [1,2,3,4]. While this system offers several advantages, it has significant limitations, such as a low drug loading capacity and expulsion from the particle over time. A modified approach to address these issues incorporates liquid lipids into the formulation, resulting in nanostructured lipid carriers (NLCs). These represent the second generation of lipid nanoparticles and have gained popularity for their ability to improve the efficacy of encapsulated drugs or active agents [5,6]. NLC systems have been extensively studied for various routes of administration, including topical, buccal, ocular, injectable, and even oral drug delivery [5,7,8,9]. The advantages of NLCs include reduced side-effects, enhanced permeation, and improved bioavailability, and they can be scaled up for industrial production [10,11]. Another advantage is that NLC systems do not require organic solvents in their preparation and can effectively encapsulate hydrophobic drugs [12]. However, applying NLCs alone to the skin, or especially within the oral cavity, can be challenging due to interference from saliva, swallowing, or food intake, which can limit their residence time at the desired site. Therefore, formulating NLCs into alternative dosage forms, such as films, by embedding them within a film matrix can enhance their performance by prolonging their retention at the site of interest and improving the therapeutic efficacy of the encapsulated drug. Additionally, incorporating nanoparticles within films can improve mechanical, thermal, and other properties [13,14,15].

**Figure 1:**
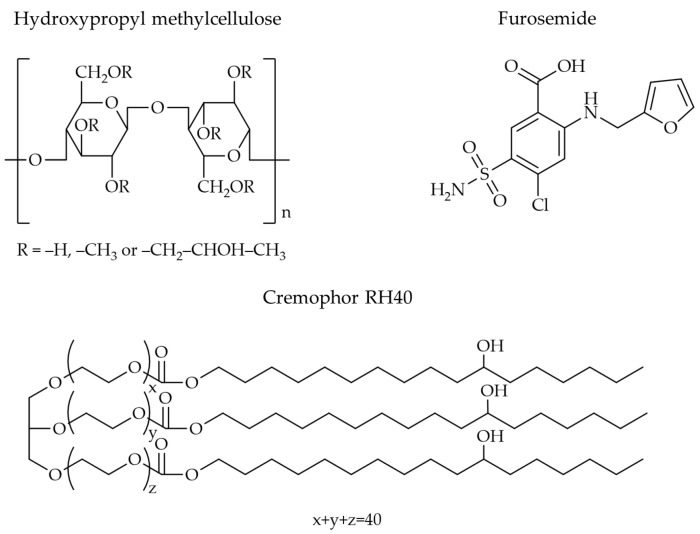
Molecular structure of HPMC, FM, and Cremophor RH40.

Films can be made from various materials, but natural polymers are widely used in research and development. Examples include chitosan, pectin, and cellulose derivatives, among which hydroxypropyl methylcellulose (HPMC) is a popular choice. The structure of HPMC consists of methoxy and hydroxypropyl groups (Figure 1) [16]. HPMC is widely used as a film-forming agent due to its ease of preparation, biocompatibility, and biodegradability [17,18,19]. Additionally, it exhibits good mucoadhesive properties on body surfaces. Many studies have investigated HPMC as a film-forming medium for nanoparticles. For example, Kraisit et al. [17] incorporated chitosan nanoparticles, and the resulting films exhibited good adhesion to the buccal mucosa and enhanced permeation of the active ingredient across the buccal mucosa for over 5 h. Tzanova et al. [20] encapsulated SLNs within HPMC films, observing an increased film thickness and flexibility, although the mechanical strength was slightly reduced. With HPMC films incorporating silver and titanium oxide nanoparticles, Khater et al. [21] reported a higher elongation at break than the film without the nanoparticles, although the latter had a higher tensile strength. Adding aluminum nanoadditives to an HPMC matrix, Mandal et al. [22] discovered that the mechanical and film barrier qualities were greatly improved. Therefore, encapsulating nanoparticles within films can improve the performance or modify the properties of both the drug and the film, leading to enhanced functionality. AlMulhim et al. [23] examined the possibility of creating a buccal film containing paliperidone-loaded nanostructured lipid carriers. They found that the pharmacokinetic profile in rabbits indicates the potential of buccal therapy, as demonstrated by a significantly higher AUC_0–12_ and greater relative bioavailability than the control. Castro et al. [24] found that when oral films and nanoparticles carried alpha-capsazepine, the permeability across both buccal and intestinal cell barriers increased when compared to film and nanoparticles alone.

Much of the current research uses trial-and-error methods to synthesize films encapsulating nanoparticles, in which one variable is held constant while the others are changed individually [25,26]. These approaches can be time-consuming and expensive and may not adequately predict the outcome, especially when there are three or more independent variables [5,19]. To address this challenge, this study employed the response surface methodology (RSM), which has the advantage of analyzing variables concurrently when complex factor interactions occur. The Box–Behnken design is the type of RSM used in this study [27]. It requires fewer runs and a shorter time for three independent variables than other designs like the central composite design [27,28].

The objective of this research was to investigate the properties of HPMC films containing NLCs loaded with furosemide (H-FM films). Furosemide (FM, Figure 1) is a BCS class IV drug, meaning it has low water solubility and low membrane permeability [29]. Furosemide-loaded NLCs (FM-NLCs) were prepared using the hot homogenization method with an ultrasonic probe for particle size reduction. The films were prepared using the casting method. Glyceryl monostearate (GMS) and castor oil were used as the solid and liquid lipids, respectively, in the preparation of NLCs because previous studies have shown that these two substances can effectively dissolve hydrophobic drugs and can be effectively combined to produce particles in the nanometer range [3,30]. Cremophor RH40 (Figure 1), a non-ionic surfactant, was used as a stabilizer in the formulation due to its low toxicity, ability to dissolve drugs, and ability to improve the stability of nanoparticles [31,32]. The particle size, size distribution (PDI), and zeta potential of the FM-NLCs dispersed in HPMC solution (H-FM dispersions) were examined before film preparation, and the effects of the three independent variables HPMC concentration (X_1_), Cremophor RH40 concentration (X_2_), and mixing speed (X_3_) were determined using a Box–Behnken design. After preparing the films containing furosemide-loaded NLCs, the physicochemical properties of the films, including weight, thickness, mechanical properties, and wettability, were evaluated.

## 2. Materials and Methods

### 2.1. Materials

Furosemide (FM) was purchased from TCI (Tokyo, Japan). Hydroxypropyl methylcellulose HPMC K4M (H-K4M) has a molecular weight of 320,000–380,000 Da, and the substituent of the methoxy group is 22% and that of the hydroxypropyl group is 8.1% [16]. H-K4M was manufactured by Dow Chemical Company (Midland, MI, USA) and kindly supplied by Rama Production Co., Ltd. (Bangkok, Thailand). Castor oil and glyceryl monostearate (GMS) were purchased from PC Drug Co., Ltd. (Bangkok, Thailand). Cremophor RH40 was purchased from Chemipan Corporation Co., Ltd. (Bangkok, Thailand), and PEG400 from Union Chemical 1986 Co., Ltd. (Bangkok, Thailand). All other chemicals in this study were of analytical grade and were used as supplied.

### 2.2. Preparation of H-FM Dispersions and Films

The film casting method was employed to fabricate H-FM films [13].

H-K4M (weight defined according to experiment design) was dissolved in distilled water (55 g), glycerin (0.6 g, used as a plasticizer) was added, and the mixture was stirred until uniform.

To prepare the FM-NLCs, GMS (1.71 g), castor oil (0.57 g), Cremophor RH40 (weight defined according to experimental design), PEG400 (0.17 g, used as a solubilizing agent), and FM (0.12 g) were combined and dissolved at 80 °C. Distilled water (~88 g) at 80 °C was added, and the biphasic mixture was stirred for 5 min at 7000 rpm using high-shear homogenization to produce the primary emulsion. The emulsion was reduced in size using a probe sonicator (Ultrasonic Processor 400, Hielscher, Teltow, Germany) set to 100% amplitude for a duration of 15 min and then allowed to cool to room temperature.

To prepare the H-FM dispersions, the H-K4M solution and FM-NLC were combined and stirred (at speeds defined according to experimental design) with a magnetic stirrer for 24 h. The particle size, zeta potential, and PDI were determined.

The H-FM dispersion (100 g) was poured onto a glass plate to prepare the H-FM films and was allowed to evaporate at RT for two days. The dried films were peeled off and stored in a vacuum desiccator until the subsequent experiments.

### 2.3. Design of Experiments and Data Analysis

A Box–Behnken design was employed to assess the impact of the three independent variables H-K4M concentration (X_1_), Cremophor RH40 concentration (X_2_), and mixing speed (X_3_) on the physicochemical characteristics of H-FM dispersions and H-FM films. The variables were tested at three different levels (+1, 0, and −1), as indicated in Table 1 (FM-1 to FM-17). The physicochemical properties of the H-FM dispersions, including particle size (Y_1_), PDI (Y_2_), and zeta potential (Y_3_), were measured. The H-FM films had several dependent variables, namely the thickness (Y_4_), weight (Y_5_), drug content (Y_6_), contact angle (Y_7_), stress (Y_8_), and elongation (Y_9_). All model formulations were analyzed using Design-Expert v9 software (Stat-Ease, Inc., Minneapolis, MN, USA). The program produced 17 random sequences comprising three levels, three factors, and five center points per block.

### 2.4. H-FM Dispersion Physicochemical Characterization

The H-FM dispersion particle size, PDI, and zeta potential were determined using a Malvern Zetasizer Nano ZS (Malvern Instruments, Malvern, Worcestershire, UK). To prevent multiple scattering, each sample was suitably diluted with distilled water and transferred into a disposable zeta cuvette.

### 2.5. Attenuated Total Reflection–Fourier Transform Infrared Spectroscopy (ATR-FTIR)

The samples were analyzed using an ATR-FTIR spectrophotometer (IRTracer-100, Shimadzu, Kyoto, Japan) to obtain their FTIR spectra. The sample was analyzed using a scanning technique with a resolution of 4 cm^−1^, covering the spectral range from 4000 cm^−1^ to 400 cm^−1^.

### 2.6. Film Weight and Thickness Measurement

H-FM films were divided into rectangular shapes with dimensions 3.5 × 0.6 cm. Film weight was accurately measured using a digital balance (model AC 210 S, Sartorius, Goettingen, Germany). Film thickness was measured using a Micrometer (STR796MXFL-25, Starrett, Athol, MA, USA).

### 2.7. Drug Content

The content of FM in films was investigated by applying a method described in the previous study [13]. Briefly, the films were cut into squares measuring 1.0 cm × 1.0 cm and placed in a test tube. Distilled water was used as the extraction solvent, with 6 mL added to the tube. At room temperature, a magnetic stirrer was used to stir the tube containing the films and solvent at 750 rpm for 24 h. The extracted FM was centrifuged at 15,000 rpm for 15 min (Model 6000, Kubota, Tokyo, Japan). After removing half a milliliter of the supernatant and filtering it through a 0.45 m cellulose membrane, the amount of FM was measured using HPLC. The HPLC (UFLC, Shimadzu, Kyoto, Japan) was equipped with a C18 column (5 μm, 4.6 × 150 mm) and a UV detector at 276 nm. The mobile phase contained phosphate buffer pH 6.8 and acetonitrile (70:30, *v*/*v*) at a flow rate of 1.0 mL/min and a retention time of 4.5 min. The FM content was determined and is presented as a percentage of the FM in the films.

### 2.8. Mechanical Properties

As described in a prior investigation [17], the mechanical properties of the H-FM films were determined with a Texture Analyzer (TA.XT.plus Texture Analyzer, Stable Micro Systems, Godalming, Surrey, UK). Maximum force and displacement values were converted to tensile strength (stress) and break elongation (strain), respectively. To calculate the parameters, the following equations were applied:

Tensile strength = F/A

where F is the maximum force required to rupture the film, A is the film’s cross-sectional area, and

Elongation (%) = ΔL/L × 100

where ΔL is the length change during the film’s break and L is the film’s initial length.

### 2.9. Film Wettability

The wettability of the H-FM films was assessed by measuring the contact angle using a sessile drop technique with a drop shape instrument (FTA 1000, First Ten Angstroms, Newark, CA, USA). The sessile drop configuration is the conventional setup for optical assessment of the contact angle through drop shape analysis. A droplet of distilled water was applied onto the surface of the film to measure the contact angle.

### 2.10. Statistical Analysis

Values were computed as the average ± standard deviation of three independent assessments (n = 3). An analysis of variance (ANOVA) was used to determine the statistical significance at the 0.05 level.

## 3. Results and Discussion

### 3.1. Physicochemical Characterization of FM-NLCs

This study investigated H-K4M films containing FM-NLCs. Before studying the film properties, it is necessary to study those of the FM-NLCs in dispersion, particularly the particle size, particle size distribution (PDI), and zeta potential. These factors provide fundamental information for further investigation of the properties of the H-FM films.

**Figure 2:**
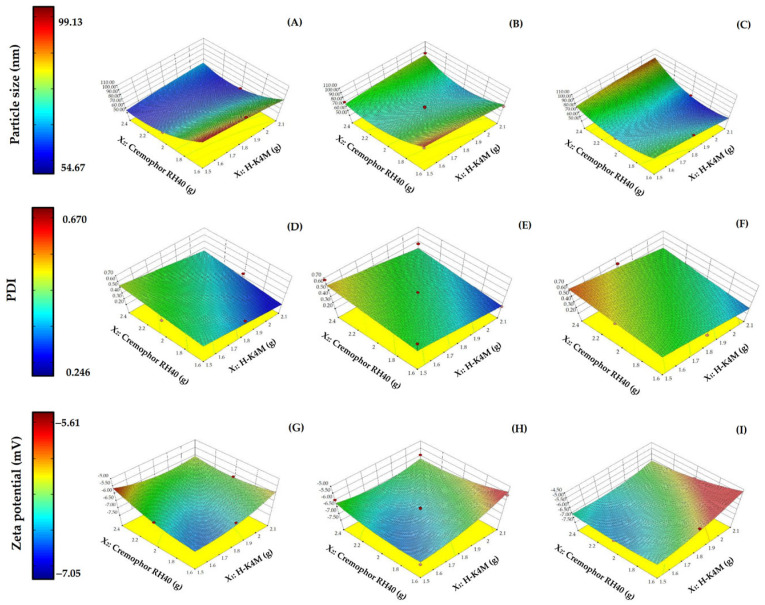
3D surfaces of FM-NLCs for particle size (**A**–**C**), PDI (**D**–**F**), and zeta potential (**G**–**I**) with mixing speeds of 700 rpm (left column), 900 rpm (middle column), and 1100 rpm (right column). (Red dots over the 3D surface refer to design points above the predicted value. Red dots under the 3D surface refer to design points below the predicted value).

The impact on the particle size of the variables mixing speed, H-K4M concentration, and Cremophor RH40 concentration are illustrated in the 3D surfaces in Figure 2A–C. The particle size varied between 54.67 and 99.13 nm. Regarding the effect of Cremophor RH40 concentration on particle size, a higher Cremophor RH40 concentration at the same H-K4M concentration and mixing speed resulted in a smaller particle size. This result agrees with several previous studies showing that increasing the amount of stabilizer leads to smaller particle sizes [3,5]. The reduction in droplet size observed in the FM-NLCs may be due to a decrease in the interfacial tension between the aqueous and oil phases facilitated by the increase in non-ionic surfactant concentration [33,34]. On the other hand, the H-K4M concentration did not affect the particle size, because H-K4M is not a primary component of the FM-NLCs. The H-K4M solution was added after FM-NLC formation to act as a film former. Higher mixing speeds of the FM-NLCs and H-K4M mixture tended to decrease the particle size. However, it is worth noting that when the concentrations of both the stabilizer and H-K4M were very high, the particles tended to be larger, especially at a speed of 1100 rpm. The area shown in the orange-red region at the top right of the 3D surface (Figure 2C) indicates that the particles were larger. The increase in particle size at high mixing speed and stabilizer and H-K4M concentrations may be caused by molecular functional group interactions or physical interactions between the polymer structure and the stabilizer [3].

The particle size distribution (PDI) ranges from 0.246 to 0.670 (Figure 2D–F). Generally, PDI values below 0.7 are considered suitable for testing with dynamic light scattering [35]. A lower PDI value indicates a narrower size distribution [36]. Increasing the Cremophor RH40 concentration did not significantly affect the PDI, while higher H-K4M concentrations tended to decrease the PDI. This is likely because H-K4M is a polymer with a large molecular weight and chain length, which can hinder the aggregation of small particles, resulting in a narrower particle size distribution. Regarding the effect of mixing speed, there may not be a clear effect on particle size with increasing mixing speed. However, at the lowest H-K4M concentration and highest Cremophor RH40 concentration, it was observed that increasing the mixing speed from 700 rpm to 1100 rpm changed the color of the 3D surface from light green to orange. This indicates that there is an increase in size distribution with increasing mixing speed under these conditions.

The zeta potentials of the FM-NLCs ranged from −7.05 to −5.61 (Figure 2G–I). All formulations had negative values, likely due to the carboxylic acid groups on the surface of the solid lipid [3,25]. Increasing the Cremophor RH40 concentration did not significantly affect the zeta potential, possibly because Cremophor RH40 is a non-ionic stabilizer. In most cases, increasing the H-K4M concentration resulted in a more positive zeta potential (change from blue area to red area), probably because the structure of H-K4M shields the negative charges on the surface of the NLCs, making them more positive (closer to zero). The effect of mixing speed on the zeta potential was unclear and depended on H-K4M and Cremophor RH40 concentrations.

**Figure 3:**
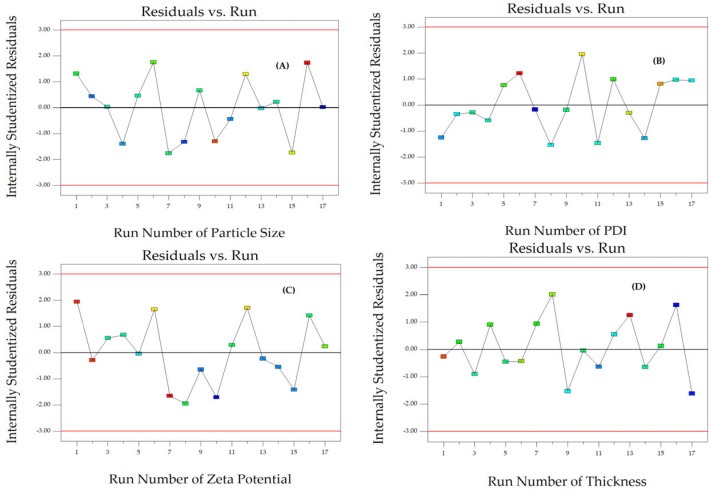
The corresponding residual plot between the run number and the internally studentized residuals for various responses of particle size (**A**), PDI (**B**), zeta potential (**C**), and thickness (**D**).

To assess the reliability of the dependent responses, we analyzed the residual plot that shows the relationship between the run number and the internally studentized residuals of Y_1_, Y_2_, and Y_3_ (Figure 3A–C). The data exhibited random scattering within a range of ±3.00, indicating that all data points were consistent with the model.

### 3.2. ATR-FTIR Spectra

**Figure 4:**
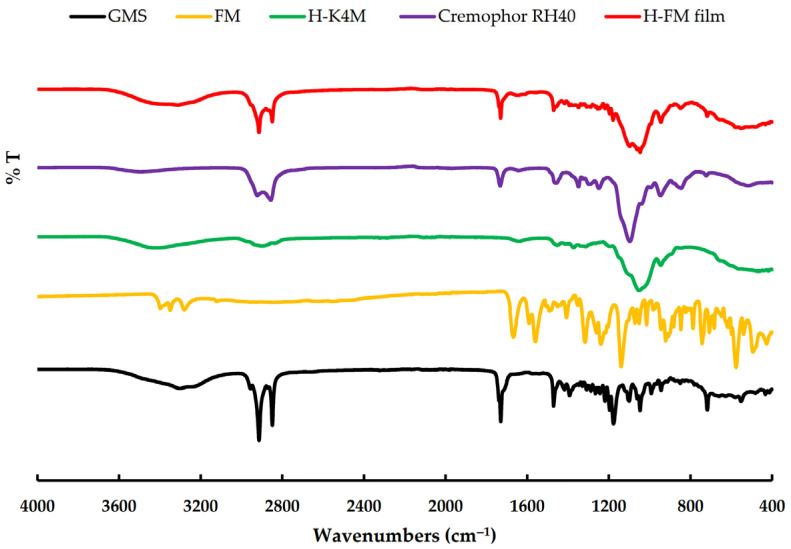
FTIR spectra of GMS, FM, H-K4M, Cremophor RH40, and H-FM film.

Figure 4 displays the FTIR spectra of GMS, FM, H-K4M, Cremophor RH40, and H-FM film. The GMS spectrum exhibited distinct peaks at approximately 3310 cm^−1^ (OH stretching), 2913 and 2848 cm^−1^ (C–H stretching), 1729 cm^−1^ (C=O stretching), and 1470 cm^−1^ (CH_3_ bending) [3,37,38]. The FM spectrum displayed major peaks at approximately 3397 and 3347 cm^−1^, corresponding to the –SO_2_NH_2_ stretching. Another peak was observed at 3275 cm^−1^, indicating N–H stretching. Additionally, peaks were observed at 1667 cm^−1^ (C=O stretching), 1588 cm^−1^ (N–H bending), 1315 cm^−1^ (R–SO_2_ symmetric stretching), 1139 cm^−1^ (S–O stretching vibrations), and 742 cm^−1^ (C–Cl stretching) [39,40,41]. The H-K4M powder spectrum exhibits distinct peaks at 3453 cm^−1^ (representing –OH stretching vibrations), 2931 cm^−1^ (indicating –CH stretching), 1452 cm^−1^ (corresponding to –CH_3_ asymmetric bending vibration), and 1051 cm^−1^ (representing –CO stretching vibration) [19,42,43]. The Cremophor RH40 spectrum exhibited obvious peaks at approximately 3502 cm^−1^ (indicating OH stretching), 2923 and 2855 cm^−1^ (representing C–H stretching), and 1730 cm^−1^ (corresponding to C=O stretching). The ether C–O–C stretching appeared as a strong broadband at 1100 cm^−1^ [44]. The FTIR spectrum of the chosen H-FM film exhibited distinct GMS peaks at approximately 2913, 2848, and 1729 cm^−1^, suggesting the presence of GMS in the films [13]. The position of the spectrum closely resembled that of the Cremophor RH40 spectrum. Thus, it demonstrated that Cremophor RH40 was a component of the H-FM film. In addition, the spectrum pattern of H-FM film at 3453 and 1051 cm^−1^ was similar to that of H-K4M. This indicated that the H-FM film included H-K4M as a film-forming agent. The absence of some FM characteristic peaks in selected H-FM film formulations could be attributed to the film containing a lower FM amount than the other components or to the complete incorporation of FM in the film matrix [3,37].

### 3.3. Film Weight, Thickness, and Drug Content

**Figure 5:**
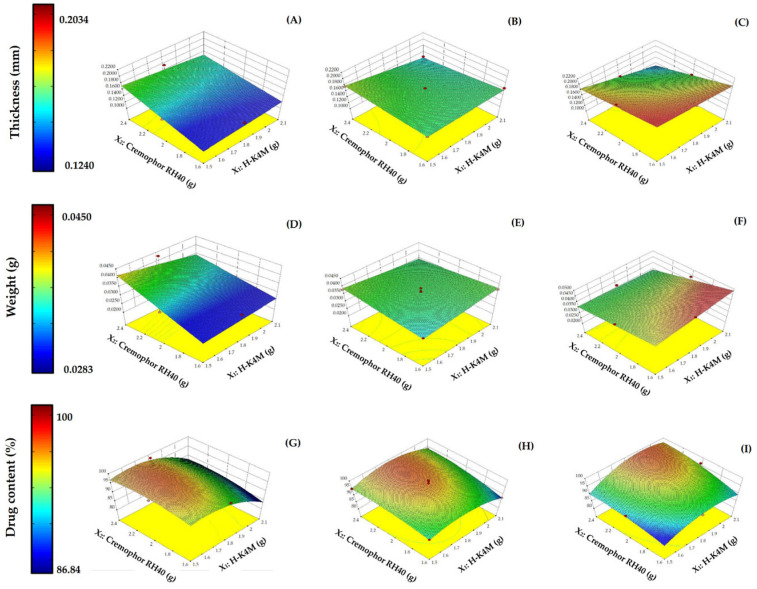
3D surfaces of H-FM films for thickness (**A**–**C**), weight (**D**–**F**), and drug content (**G**–**I**) with mixing speeds of 700 rpm (left column), 900 rpm (middle column), and 1100 rpm (right column). (Red dots over the 3D surface refer to design points above the predicted value. Red dots under the 3D surface refer to design points below the predicted value).

The impact on the film thickness of the variables mixing speed, Cremophor RH40 concentration, and H-K4M concentration is shown in the 3D relationship surface in Figure 5A–C. The thickness of the prepared films ranged from 0.1240 to 0.2034 mm. Increasing the Cremophor RH40 concentration to a certain level at a mixing speed of 700 rpm and a constant H-K4M concentration (Figure 5A) increased the thickness of the film. However, at 900 rpm (Figure 5B), the thickness of the film was not significantly affected; at 1100 rpm (Figure 5C), the thickness decreased with increasing Cremophor RH40 concentration. However, the change in film thickness due to the increased Cremophor RH40 concentration was insignificant. Increasing the mixing speed tended to significantly increase the film thickness (*p* < 0.05), especially when the concentrations of both Cremophor RH40 and H-K4M were low. Increasing the H-K4M concentration did not significantly affect the film thickness. Therefore, the factor that affected the thickness of the prepared film was the mixing speed. An appropriate speed allows the FM-NLCs and H-K4M solution to mix well, resulting in a film that is not too thick. However, the two substances may not mix well if the speed is too high, resulting in a thick film, although this also depended on the Cremophor RH40 and H-K4M concentrations.

The impact on the film weight of the three variables is shown in Figure 5D–F. The H-FM films weighed between 0.0283 and 0.0450 g. The 3D surface patterns at mixing speeds of 700 and 900 rpm (Figure 5D,E) were like those for film thickness at the same mixing speeds (Figure 5A,B). At 700 rpm, increasing the Cremophor RH40 concentration tended to increase the weight, while increasing the H-K4M concentration did not significantly affect the weight at the same concentration of Cremophor RH40. However, at 900 rpm, increasing both Cremophor RH40 and H-K4M concentrations had almost no effect on the film weight. At 1100 rpm (Figure 5F), the weight of the film increased when the concentrations of H-K4M and Cremophor RH40 were high and low, respectively. However, when the amount of Cremophor RH40 was greatly increased, the film weight decreased. Nevertheless, the mixing speed was still the main factor affecting both the film weight and thickness.

Figure 5G–I illustrate the influence of the three variables on the percentage of drug content. The percentage of drug content in H-FM film ranged from 86.84 to 100. The high percentage of drug content indicated that no FM was lost or it lost in small amounts during the film preparation process. At a mixing speed of 700 rpm (Figure 5G), it was found that increasing the amount of H-K4M decreased the drug’s percentage. However, at low amounts of H-K4M, the drug’s percentage was high. Cremophor RH40’s effect on the percentage of drugs was dependent on the amount of H-K4M. At a mixing speed of 900 rpm (Figure 5H), neither HK4M nor Cremophor RH40 amounts clearly affected the percentage of drug content. At a mixing speed of 1100 rpm (Figure 5I), it was found that when the amount of HK4M and Cremophor RH40 was low, the percentage of the drug was also low. On the other hand, if the amount of both substances was high, the percentage of the drug amount would also be high. Consequently, the percentage of drug content is influenced by three factors, the amount of H-K4M, Cremophor RH40, and mixing speed, as previously mentioned. The appropriate selection of these factors will ensure that a high percentage of the drug is encapsulated in the film.

**Figure 6:**
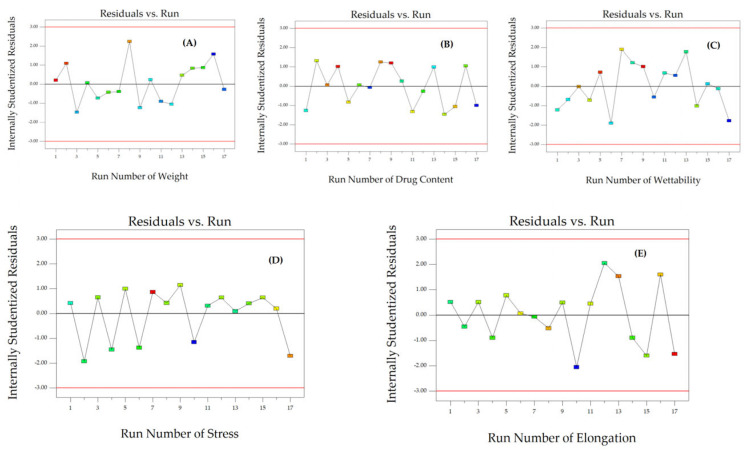
The corresponding residual plot between the run number and the internally studentized residuals for various responses of weight (**A**), drug content (**B**), wettability (**C**), stress (**D**), and elongation (**E**).

The reliability of Y_4_, Y_5_, and Y_6_ was assessed by comparing the run number with the internally studentized residuals (Figure 3D and Figure 6A,B). The randomly scattered data points within the specified limits indicate that all data were accurately aligned with the model.

### 3.4. Wettability and Mechanical Properties

**Figure 7:**
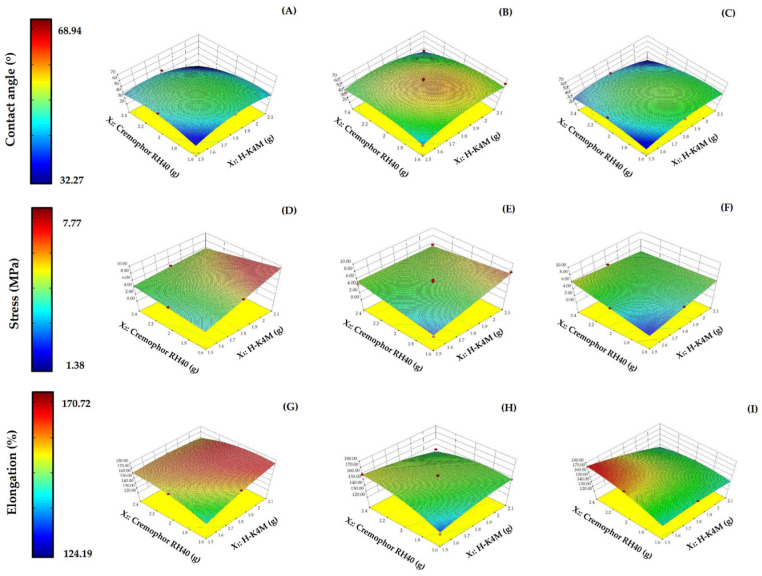
3D surfaces of H-FM films for contact angle (**A**–**C**), stress (**D**–**F**), and % elongation (**G**–**I**) with mixing speeds of 700 rpm (left column), 900 rpm (middle column), and 1100 rpm (right column). (Red dots over the 3D surface refer to design points above the predicted value. Red dots under the 3D surface refer to design points below the predicted value).

The surface properties of the H-FM films were analyzed in terms of contact angle to determine their wettability and hydrophilicity. The contact angle of the H-FM films ranged from 32.27 to 68.94° (Figure 7A–C). The contact angle of a water droplet can indicate the wettability of a material. If the value is high, the sample has low wettability [45]. Wettability is used to study other properties, such as dissolution, mucoadhesion, and swelling. In this study, increasing the mixing speed or the concentrations of Cremophor RH40 or H-K4M did not clearly affect the contact angle. However, the contact angles for all formulations were less than 90°, indicating that the films were hydrophilic [46].

In terms of the mechanical properties of the film, the tensile strength and elongation were investigated. These properties indicate the film’s resistance to force or tearing. The tensile strength of the films ranged from 1.38 to 7.77 MPa (Figure 7D–F), and the elongation ranged from 124.19 to 170.72% (Figure 7G–I). Increasing the Cremophor RH40 concentration did not show a clear trend in the change in tensile strength. However, increasing the H-K4M concentration significantly increased the tensile strength (*p* < 0.05), especially when the Cremophor RH40 concentration was low at mixing speeds of 700 and 900 rpm (Figure 7D,E). The enhanced tensile strength achieved by incorporating H-K4M can be attributed to an augmented formation of intermolecular hydrogen bonds between the OH groups of the H-K4M backbone and the OH and COO– groups of GMS or other chemical compounds [13,47]. These interactions can enhance the film’s tensile strength, particularly when the Cremophor RH40 concentration is low. At higher surfactant concentrations, the space between adjacent H-K4M chains increases, decreasing the structural integrity and tensile strength of the film [48]. The effect of mixing speed on the tensile strength trend was unclear and dependent on the formulation’s Cremophor RH40 and H-K4M concentrations.

The elongation value, which indicates the flexibility of the film, increased at high Cremophor RH40 and low H-K4M concentrations, especially at mixing speeds of 700 and 1100 rpm. A high surfactant concentration alone led to a decrease in elongation, possibly due to the disturbance of the films’ structural integrity [13]. The impact on elongation of either H-K4M concentration or mixing speed alone was unclear, depending also on the other influencing variables.

The reliability of Y_7_, Y_8_, and Y_9_ was assessed by comparing the internally studentized residuals to the run number (Figure 6C–E). The randomly dispersed data points within the limits indicate that all the data were adequately fitted with the model.

## 4. Conclusions

This study employed a Box–Behnken design to assess the impact of three main independent variables, namely H-K4M concentration (X_1_), Cremophor RH40 concentration (X_2_), and mixing speed (X_3_), on the physicochemical characteristics of FM-NLCs and H-FM films. The three independent factors impacted the particle size, PDI, and zeta potential of FM-NLCs, which were nanometer-sized, narrowly distributed, and negatively charged. The physical properties of the films, such as thickness, weight, wettability, and mechanical properties, were also impacted by these variables. The high percentage of drug content indicated that no FM was lost or it was lost in small amounts during the film preparation process. The ATR-FTIR analysis confirmed the drug’s complete incorporation into the film matrix. The contact angles of all film formulations were below 90°, indicating that the films possessed hydrophilic properties. Increasing the concentration of H-K4M significantly improved the tensile strength, especially when the Cremophor RH40 concentration was low. The interactions between H-K4M, GMS, and other chemicals can potentially increase the film’s tensile strength, especially when the Cremophor RH40 concentration is low. However, the elongation value increased when the Cremophor RH40 concentration was increased at low H-K4M concentrations, especially at mixing speeds of 700 and 1100 rpm. This phenomenon can be attributed to the disruption of the structural integrity of the films due to a high surfactant concentration. Hence, the three variables play a crucial role in determining the properties of the prepared films. This information is valuable for determining the optimal values for variables when developing films for future use in drug delivery systems. Furthermore, the glyceryl monostearate and castor oil ratio is an interesting consideration for improving the properties of NLCs and films.

## Figures and Tables

**Table 1:** Box–Behnken experimental design and independent variables.

Formulation	Actual Factors of Independent Variables			Coded Factors of Independent Variables			
	H-K4M (g), X_1_	Cremophor RH 40 (g), X_2_	Mixing Speed (rpm), X_3_	Levels	H-K4M (g), X_1_	Cremophor RH 40 (g), X_2_	Mixing Speed (rpm), X_3_
FM-1	1.8	1.6	1100	−1	1.50	1.60	700
FM-2	2.1	2	1100	0	1.80	2.00	900
FM-3	1.8	2	900	+1	2.10	2.40	1100
FM-4	1.8	2	900				
FM-5	1.8	2	900				
FM-6	1.5	2.4	900				
FM-7	2.1	1.6	900				
FM-8	1.8	2.4	700				
FM-9	1.8	2	900				
FM-10	1.5	1.6	900				
FM-11	1.5	2	700				
FM-12	2.1	2.4	900				
FM-13	1.5	2	1100				
FM-14	1.8	2	900				
FM-15	1.8	2.4	1100				
FM-16	1.8	1.6	700				
FM-17	2.1	2	700				

## Data Availability

Data will be made available on request.
